# Comparative Whole Genome Phylogeography Reveals Genetic Distinctiveness of Appalachian Populations of Boreal Songbirds

**DOI:** 10.1111/eva.70163

**Published:** 2025-10-15

**Authors:** Abigail A. Kimmitt, Teresa M. Pegan, Kristen S. Wacker, Andrew W. Jones, Benjamin M. Winger

**Affiliations:** ^1^ Department of Ecology and Evolutionary Biology and Museum of Zoology University of Michigan Ann Arbor Michigan USA; ^2^ Department of Biology Hofstra University Hempstead New York USA; ^3^ Department of Organismic and Evolutionary Biology Harvard University Cambridge Massachusetts USA; ^4^ Department of Ornithology Cleveland Museum of Natural History Cleveland Ohio USA; ^5^ Spring Island Trust Okatie South Carolina USA

**Keywords:** comparative population genomics, genetic diversity, molecular parallelism, seasonal migration

## Abstract

Intraspecific genetic diversity across a species' geographic range is relevant to adaptive potential and long‐term population persistence, and identifying genetically distinct groups within species can direct management decisions focused on conserving species‐level genetic diversity. Comparative phylogeography using whole genome techniques allows for investigation of whether co‐distributed species exhibit shared spatial genetic differentiation at fine spatial scales, thereby facilitating a comparative approach to both landscape and conservation genetics. By sequencing over 900 low‐coverage whole genomes, we evaluated the concordance of genetic structure and diversity from 12 co‐occurring species of migratory birds whose breeding ranges span adjacent North American ecogeographic regions: the vast boreal forest belt and the temperate montane Appalachian forests. We detected concordant phylogeographic patterns in 11 of 12 species wherein populations from the southern Appalachians were genetically distinct from boreal belt populations. Our results reveal that small populations persisting in the southern Appalachian Mountains consistently harbor genetic diversity that is subtly distinct from much larger, widespread boreal populations of the same species. However, in most species, levels of standing genetic diversity were not significantly different between Appalachian and boreal populations despite the drastic difference in geographic extent of these populations. We found no evidence for shared signatures of selection across the genome, suggesting that the concordance of spatial genetic structure across species emerges from species‐specific patterns of molecular divergence across the genome rather than parallel patterns of selection. Conservation of the Appalachian ecosystem would likely support maintenance of distinct genetic diversity in several migratory avian species with widespread distributions.

## 1 Introduction

**FIGURE 1:**
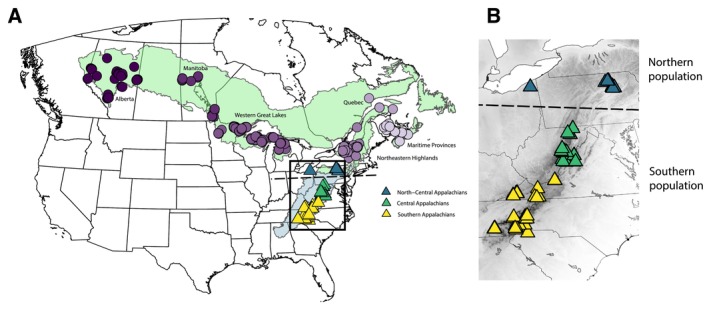
(A) Map of specimen sampling locations for all 12 species. We sampled an average of 75.6 individuals (range = 35–94) per species across the full Boreal‐Appalachian breeding range. Each point represents an individual, but in some cases, multiple individuals were sampled from the same location, such that points are overlapping. The boreal forest (green) and the Appalachian forests (light blue) were designated following “level 1” and “level 2” ecoregions, respectively defined by Omernik and Griffith (2014). Samples from the boreal region are shown in purple, with shading corresponding to longitude. (B) The inset map focuses on the three sampling regions associated with the Appalachian Mountains south of the boreal forest belt: (1) North‐Central Appalachians (teal), (2) Central Appalachians (green), (3) Southern Appalachians (yellow). Elevation is represented in grayscale, with darker colors showing higher elevation. In both maps, the dotted line demarcates the geographic boundary between “Northern” and “Southern” populations that exhibited genetic differentiation in 11 of 12 study species (Figure 2).

As a “place‐based” field, comparative phylogeography investigates the geographic context of genetic variation and population structure in co‐occurring species (Avise et al. 1987; Edwards et al. 2022; Knowles 2009). The development of cost‐effective whole genome techniques has created a new era of comparative phylogeography wherein researchers can evaluate spatial genetic differentiation at high resolution among many co‐distributed species, thereby facilitating landscape and conservation genetics at a comparative scale (Grueber 2015; McGaughran et al. 2022; Rissler 2016). Here, we use whole genome sequencing to investigate the concordance of genetic structure and diversity patterns in co‐occurring migratory birds whose breeding ranges span adjacent ecogeographic regions: the vast boreal forest belt that extends longitudinally across northern North America and a connected narrow peninsula of montane habitat that runs south along the Appalachian Mountains (Figure 1). Specifically, we evaluated 900 genomes from 12 bird species to test whether Appalachian populations are genetically distinct from the considerably larger boreal belt populations, whether they have consistent population differences in genetic diversity, and whether parallel patterns of molecular evolution underlie population differentiation in these co‐distributed species.

Many boreal plants and animals have broad ranges that extend across northern North America, with relict populations persisting further south in similar habitats provided by montane temperate forests (Campbell et al. 2021; Wiken et al. 2011). The southern and central Appalachian Mountains are at a lower latitude than the boreal forest belt and cover a much smaller geographic area, but support mixed deciduous‐coniferous montane forest that is suitable for many boreal forest species of birds (Kimmitt et al. 2024a; Leppold and Mulvihill 2011; Miller et al. 2024) and other taxa (Campbell et al. 2021; Mitchell et al. 1997) (Figure 1). Phylogeographic structure between boreal and Appalachian populations could be driven by historic refugial species distributions or the unique ecology that distinguishes the southern and central Appalachians from the northern boreal forest belt (Omernik and Griffith 2014), with differences in biogeographic history, climate, and forest structure (Cogbill and White 1991; Lyman and Edwards 2022; Wiken et al. 2011). However, species with high dispersal ability are expected to exhibit low genetic differentiation (Claramunt et al. 2012; Medina et al. 2018), such that we might expect that migratory birds would exhibit panmixia or subtle patterns of isolation by distance, rather than discrete differentiation between the boreal and Appalachian ecoregions. A recent population genetic analysis of a migratory boreal avian assemblage of 35 co‐distributed species found that species vary in their spatial population genetic patterns across the North American boreal forest belt, ranging from panmixia to significant isolation by distance (Pegan et al. 2025b). However, the degree of genetic divergence between widespread boreal and geographically restricted Appalachian populations of migratory birds remains understudied at a comparative scale. Here, by incorporating additional sampling for 12 species from Pegan et al. (2025b) whose ranges extend south into the Appalachian montane forests, we evaluate whether Appalachian populations exhibit variable or shared phylogeographic patterns in relation to the geographically more expansive boreal forest belt.

The effects of habitat loss and climate change on montane taxa are pressing conservation issues globally (Freeman et al. 2018, 2021), as well as locally in the Appalachian region (Lewis et al. 2023; Milanovich et al. 2010; Potter et al. 2010). Populations of boreal birds in the southern peripheries of their ranges, including Appalachia, are likely at higher extirpation risk than boreal belt populations due to the smaller geographic area paired with acute anthropogenic pressure (Leppold and Mulvihill 2011; Lituma et al. 2021; Mitchell et al. 1999; Ralston and DeLuca 2020). A net loss of approximately 2.5 billion migratory North American birds since 1970 (Rosenberg et al. 2019), and climate‐induced range shifts forcing birds to higher latitudes (McCaslin and Heath 2020) further highlight the need to understand genetic diversity in southern Appalachian populations. Knowledge of Appalachian populations' genetic diversity and connectivity with larger boreal populations is relevant to their conservation management, as genetic diversity across a species' range is critical for long‐term population persistence and adaptive potential (Exposito‐Alonso et al. 2022; Laikre et al. 2020). Therefore, in addition to testing for genetic structure, we evaluated intraspecific variation in levels of population genetic diversity in boreal versus southern Appalachian populations in each of the study species.

Specifically, we tested whether the geography of genetic diversity in each species supports alternative hypotheses that derive from the effects of historic versus contemporary processes on population demographic trajectories. Much of the contemporary boreal forests was covered by ice during the Last Glacial Maximum, whereas the southern and central Appalachians were south of the Laurentide Ice Sheet (Lyman and Edwards 2022). This pattern has led to the suggestion that the Appalachian Mountains served as a glacial refugium from which populations expanded into the boreal forests after the ice sheet retreated (Lyman and Edwards 2022; Soltis et al. 2006), a hypothesis that predicts higher levels of genetic diversity in contemporary southern (Appalachian) populations than northern (boreal forest) populations (Excoffier 2004; Hewitt 1999; Provan and Bennett 2008; Ralston et al. 2021). However, an alternative hypothesis deriving from modern differences between ecoregions in geographic range and census population sizes (Lyman and Edwards 2022; Soltis et al. 2006) predicts the opposite patterns in genetic diversity, wherein Appalachian populations may exhibit lower genetic diversity than the larger boreal population. Assessing relative levels of genetic diversity in boreal versus Appalachian populations will elucidate the relevance of population genetics to conservation of Appalachian diversity.

Whole genome phylogeographic sampling creates the opportunity not only to assess spatial population genetic patterns, but also to evaluate the genomic context of these patterns. For example, genomic divergence can reflect effects of few loci with very strong structure (e.g., Sodeland et al. 2016; Toews et al. 2016; Harringmeyer and Hoekstra 2022), or the subtle effects of many loci (e.g., Novembre et al. 2008; Michel et al. 2010; Sendell‐Price et al. 2020). Therefore, after determining patterns of spatial genetic structure in each species, we examined whether genetic differentiation tended to be diffuse or localized in the genome. Positive selection in a population, or local adaptation, is expected to drive large differences in allele frequencies in localized positions on the genome, whereas neutral evolutionary processes are more likely to result in differentiation that is more widely distributed throughout the genome (Lewontin and Krakauer 1973; Hahn 2019). If genetic structure in species is primarily driven by neutral phylogeographic processes, we expect that differentiation should be distributed diffusely across the genome. Alternatively, if local adaptation to environmental differences between divergent populations drives genetic structure within species, we expect more localized peaks of genomic differentiation around loci responsible for the selected traits. Finally, where we observed concordant population structure in closely related species, we tested for parallel patterns of genomic differentiation, that is, evidence that the same genomic regions drive genetic differentiation across multiple species. Although scans for molecular parallelism are more typically used to determine the molecular basis of known phenotypic convergence (Manceau et al. 2010; Lee and Coop 2019; Sackton and Clark 2019), we apply this approach to investigate whether parallel genomic adaptation (e.g., shared candidate genes between co‐distributed species) might underlie congruent phylogeographic patterns of genetic variation.

## 2 Methods

### 2.1 Study System and Sampling

We examined population structure in 12 co‐distributed bird species for which comprehensive tissue sampling was available across the breeding range, spanning the boreal forest belt and the Appalachian Mountains (Table S1). Our study system includes one woodpecker species (Piciformes), while the remaining species are from seven families of songbirds (Passeriformes) (Table 1). Species differ in migratory distance, but otherwise have similar life histories (e.g., mating system, age to first breeding season) and general habitat associations (mixed coniferous‐deciduous forest) (Winger and Pegan 2021). Sampling across the boreal forest belt extended from Alberta through the western Great Lakes and northeastern highlands of the United States to the Maritime provinces (Figure 1, Figure S1). In the northeast, our boreal belt sampling region includes parts of the northern Appalachian Mountains as geologically defined (e.g., Green Mountains of Vermont), but our reference to Appalachian sampling refers specifically to the peninsula of montane temperate forests extending south of the boreal belt (Figure 1). We refer to these Appalachian sampling regions as the north‐central Appalachians (Pennsylvania and Ohio; teal triangles, Figure 1), the central Appalachians (West Virginia, Maryland, and northern Virginia; green triangles, Figure 1), and the southern Appalachians (North Carolina and southern Virginia; yellow triangles, Figure 1).

**TABLE 1:** Whole genome analyses include population differentiation (*F*_ST_) and population‐level genetic diversity (mean ± standard deviations of pairwise *θ*_*π*_ calculated across the chromosomes) for each species.

Family	Species	Whole genome					Mitochondrial				
		*N*	*F*_ST_	Pairwise *θ*_*π*_			*N*	*F*_ST_	Nei's *D*	Nucleotide diversity (Southern/Northern)	Tajima's *D*
		*N*_Southern_/*N*_Northern_		Southern (mean ± SD)	Northern (mean ± SD)	*p*	*N*_Southern_/*N*_Northern_				
Picidae	*Sphyrapicus varius*	3/63	0.006	—	—	—	3/63	—	—	—/0.003	−2.42
Vireonidae	*Vireo solitarius*	28/56	0.006	0.0102 ± 0.0013	0.0069 ± 0.0013	**< 0.0001**	27/53	0.014	0.004	0.004/0.003	−2.02
Regulidae	*Regulus satrapa*	28/50	0.004	0.0075 ± 0.0019	0.0068 ± 0.0019	**0.0002**	28/50	0.000	0.001	0.001/0.001	−2.66
Certhiidae	*Certhia americana*	10/48	0.004	0.0052 ± 0.0011	0.0050 ± 0.0009	0.3806	10/48	0.076	0.012	0.002/0.002	−2.07
Troglodytidae	*Troglodytes hiemalis*	9/26	0.005	0.0062 ± 0.0018	0.0060 ± 0.0016	0.0818	1/24	—	—	—/0.029	−2.31
Turdidae	*Catharus fuscescens*	29/65	0.008	0.0157 ± 0.0035	0.0159 ± 0.0038	0.6903	29/64	0.001	0.002	0.002/0.003	−2.20
Turdidae	*Catharus guttatus*	5/63	0.005	—	—	—	5/63	—	—	—/< 0.001	−2.42
Passerellidae	*Junco hyemalis*	12/58	0.014	0.0063 ± 0.0007	0.0063 ± 0.0006	0.4994	6/64	0.086	0.017	0.001/0.001	−1.68
Parulidae	*Setophaga coronata*	9/71	0.004	0.0093 ± 0.0009	0.0094 ± 0.0008	0.3184	9/68	0.005	0.005	0.002/0.002	−2.06
Parulidae	*Setophaga fusca*	20/51	0.001	0.0122 ± 0.0009	0.0113 ± 0.0007	**< 0.0001**	20/51	0.012	0.002	0.002/0.001	−2.65
Parulidae	*Setophaga magnolia*	22/68	0.006	0.0121 ± 0.0008	0.0122 ± 0.0008	0.5364	22/60	0.055	0.005	0.002/0.001	−2.34
Parulidae	*Setophaga virens*	25/68	0.003	0.0056 ± 0.0005	0.0077 ± 0.0004	**< 0.0001**	25/65	0.029	0.006	0.005/0.005	−1.75

*Note:* Number of individuals used for calculating whole genome and mitochondrial statistics are reported as *N*_Southern_/*N*_Northern_. We included *p*‐values from a Wilcoxon‐rank sum test used to compare mean pairwise *θ*_*π*_ between Southern and Northern populations. For comparison to traditional mitochondrial analyses, ND2 statistics include population differentiation (*F*_ST_, Nei's *D*), population‐level genetic diversity (nucleotide diversity), and species‐level Tajima's *D*. We excluded *S. varius* and *C. guttatus* from pairwise *θ*_*π*_ analyses given the very small sampling size of the Southern population (*n* ≤ 5 individuals). We also excluded *S. varius* , *C. guttatus* , and *T. hiemalis* from Southern genetic diversity measures and the two measures of population differentiation, due to very low numbers of sequenced mitochondrial genomes (*n* ≤ 5 individuals). Significance of *p* < 0.05 is denoted in bold.

We included in our study 656 previously sequenced low‐coverage genomes from across the boreal belt from a study focused on genetic structure within the boreal belt region (Pegan et al. 2025b) and 47 samples from the Appalachian range of one species, *Catharus fuscescens* , from a range‐wide phylogeographic study (Kimmitt et al. 2024a). Following an identical protocol as these studies, we sequenced an additional 216 samples from the three Appalachian sampling regions of the remaining 11 species to achieve sampling in both the boreal and Appalachian ranges of these species. All DNA sequenced for this manuscript was extracted using DNeasy Blood and Tissue Kits (Qiagen Sciences, Germantown, MD, USA) from frozen or ethanol‐preserved tissue samples from existing natural history collections or blood samples that were collected during the breeding season (Table S1, see Acknowledgments). We prepared libraries for low‐coverage sequencing using a modified Illumina Nextera protocol (Schweizer and DeSaix 2023; Therkildsen and Palumbi 2017) and then sequenced using paired‐end sequencing of 150 bp reads. Libraries were sequenced on an Illumina HiSeq, NovaSeq 6000, or NovaSeq X platform.

### 2.2 Data Processing

Following the methods used by Pegan et al. (2025b) and Kimmitt et al. (2024a), we trimmed adapters and low‐quality bases from demultiplexed data using the –trimns and –trimqualities options in AdapterRemoval v2.3.1 (Schubert et al. 2016). To reduce potential sequencing batch effects, we removed low‐quality read ends using fastp v0.23.2 (Chen et al. 2018) with the ‐‐cut_right option (Lou and Therkildsen 2022).

We assembled mitochondrial genomes for each sample using NOVOplasty (v. 4.3.1) (Dierckxsens et al. 2017). Using at least one mitochondrial gene from each sample, we used BLAST in Geneious (v. 2021.2.2) to confirm species identity of each sample and check for evidence of cross‐contamination and hybridization during extractions and/or library preparation (as described in Kimmitt et al. 2023, 2024a). We removed 12 samples that failed to sequence, showed evidence of cross‐contamination, or showed evidence of species misidentification.

We aligned samples from each species in our dataset to a closely related species with an available chromosome‐level reference genome (Table S2; Toews et al. 2016; Manthey et al. 2021; Friis et al. 2022) using bwa mem (Li and Durbin 2010) and Samtools (Li et al. 2009). We removed overlapping reads using clipOverlap in bamUtil (Jun et al. 2015), marked duplicate reads with MarkDuplicates, and assigned all reads to a new read group with AddOrReplaceReadGroups using picard (http://broadinstitute.github.io/picard/). We then indexed all bam files using Samtools (Li et al. 2009). We discarded one sample ( *Troglodytes hiemalis* ) given low mapping quality (< 50%). We aligned samples around indels using GATK (v. 3.7) (Van der Auwera et al. 2013) by applying RealignerTargetCreator to the entire dataset and using IndelRealigner for each sample.

After filtering, we included a total of 906 samples in the final analyses. Genotype likelihoods from low‐coverage sequencing data were calculated using the GATK model in ANGSD v0.9.40 (Korneliussen et al. 2014). We analyzed results using a genotype likelihood framework given the genotype uncertainty associated with low‐coverage sequencing (Korneliussen et al. 2014; Lou et al. 2021). The options used for ANGSD analyses are detailed in Table S3.

### 2.3 Population Structure

**FIGURE 2:**
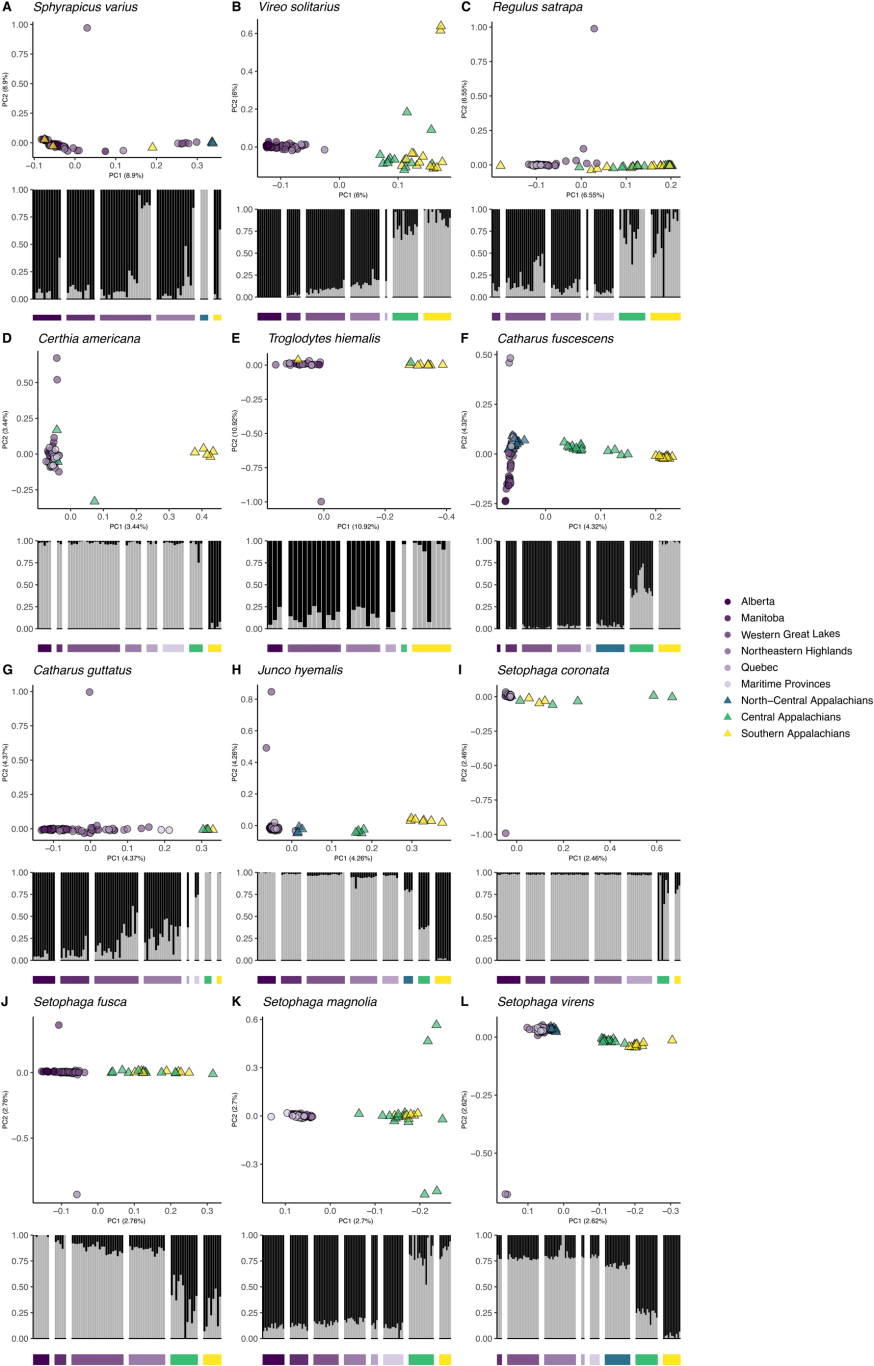
Principal Component Analysis (PCA) (top) and admixture plots (*K* = 2) (bottom) for each species. Scientific name is at the top of each panel. Colors represent sampling location across the Boreal‐Appalachian range. We denote Appalachian sampling regions as triangles, and boreal belt sampling regions as circles with purple shading corresponding to longitude in Figure 1. All species exhibit spatial patterns of genetic differentiation associated with a phylogeographic break in the central or southern Appalachians, except for *Sphyrapicus varius* (panel A).

We used ANGSD to calculate genotype likelihoods for all sites across the genome that were determined to be polymorphic (i.e., SNPs) at an alpha level of 0.05 (Korneliussen et al. 2014). We filtered paralogous or mis‐mapped SNPs out of the dataset using ngsParalog v1.3.2 (https://github.com/tplinderoth/ngsParalog; Linderoth 2018), a program that implements a likelihood method to detect mapping problems specifically in low‐coverage sequencing data. To visually assess the spatial population genetic structure of each species, we conducted Principal Component Analyses (PCA) with PCAngsd v1.10 (Meisner and Albrechtsen 2018). Because genomic inversions can obscure geographic structure in PCAs (Novembre et al. 2008; Tian et al. 2008; Novembre and Peter 2016), we used visualizations from single‐chromosome PCAs and lostruct (Li and Ralph 2019) to detect putative inversions across all chromosomes and all 12 species as implemented in PCAngsd (https://github.com/alxsimon/local_pcangsd). We removed blocks of macrochromosomes and full microchromosomes with evidence of inversions from the dataset (Table S4). All sex chromosomes were also removed from the dataset because the quality and the sex of the reference genomes varied, which could introduce bias into our analyses. After filtering out all inversions, we produced genome‐wide PCAs and admixture plots using the ‐‐admix option in PCAngsd to both determine the best fit for the number of ancestry populations (i.e., *K*) as well as to estimate admixture proportions for that *K* using a non‐negative matrix factorization algorithm. Differentiation on PC2 in *Junco hyemalis* in the genome‐wide PCA was associated with sex. Since we could not account for the cause of this pattern, we removed 19 females from the final PCA as well as all other whole genome analyses. In some species, PCA revealed outliers along the non‐geographic PC axis (PC 2), for which the source of variation is unknown. Removal of these outliers produced subsequent outliers without changing the nature of the genetic structure associated with geography. Therefore, we have retained all points in the PCA figures that had not been removed for previously described reasons (Figure 2).

We calculated genome‐wide pairwise *F*_ST_ between two distinct genetic populations that emerged from the admixture analyses and visually in our PCAs in most species (see Section 3 and Figures 1 and 2): (1) a “Northern” population that included the boreal belt and the north‐central Appalachians and (2) a “Southern” population that included the southern and central Appalachians (Figure 1). Generation of site frequency spectra with genotype likelihood data is computationally intensive, so we created a subset of loci for each reference genome, including both variant and invariant sites, by randomly subsampling stretches of 2 kb loci at least 10 kb apart (yielding about 10% of the whole genome), using scripts modified from https://github.com/markravinet/genome_sampler. We removed all sites flagged by ngsParalog from the subsampled dataset and stored the loci in a BED file. Using the ‐doSaf command and the ‐sites filter with our BED file, we generated a site allele frequency (SAF) file in ANGSD for these subsampled loci. We used winsfs (Rasmussen et al. 2022) to create folded two‐dimensional (2D) site frequency spectra (SFS) between each population pair. We then used the *F*_ST_ index and stats function with the option ‐whichFst 1 (the Bhatia estimator, which is recommended for uneven sample sizes; Bhatia et al. 2013) in ANGSD to calculate global pairwise *F*_ST_.

### 2.4 Genetic Diversity

For population‐level genetic diversity measures (pairwise *θ*_*π*_), we used the same genetic populations (Northern and Southern) described above and calculated genetic diversity measures using site allele frequencies and site frequency spectra. We used the same subsampled and filtered set of loci including invariant sites, as previously described. For all 12 species, the sample size across the Northern population was much larger than the sample size for the Southern population, reflecting the differences in geographic size of the landscape covered by each population. Therefore, for each species, we created five randomly subsampled groups of individuals from the Northern population, each matching the sample size of that species' Southern population. For each species, we then generated population‐level site allele frequency (SAF) files in ANGSD with the ‐doSaf parameter and excluded sites flagged by ngsParalog and putative inversions. We generated population‐level 1‐dimensional (1D) site frequency spectra (SFS) using winsfs (Rasmussen et al. 2022). We calculated pairwise *θ*_*π*_ for each chromosome using the saf2theta and thetaStat functions in ANGSD. For the Northern populations, we repeated these analyses individually on the five random subgroups for each species and then averaged per‐chromosome *θ*_*π*_ among these subgroups. We compared each species' average Northern population pairwise *θ*_*π*_ to the Southern population's pairwise *θ*_*π*_ using a nonparametric Wilcoxon rank‐sum test. We excluded *Sphyrapicus varius* and *Catharus guttatus* from pairwise *θ*_*π*_ analyses given the very small sampling size of the Southern population (*n* ≤ 5 individuals).

### 2.5 Mitochondrial Analyses

To put our whole genome results in the context of a long history of previous avian comparative phylogeographic analyses from other regions that relied on mitochondrial genes (e.g., Zink 1996; Klicka and Zink 1997; Smith et al. 2014; Winger and Bates 2015), we also measured key population statistics using the mitochondrial gene ND2 across all 12 species, which was a byproduct of the whole genome sequencing (described above). We calculated the average pairwise fixation index as well as Nei's distance between the Southern and Northern populations using R packages adegenet v 2.1.5 (Jombart 2008) and hierfstat v 0.5‐10 (Goudet 2005). For population comparisons in our mitochondrial analyses (*F*_ST_ and Nei's *D*), we excluded *C. guttatus* , *S. varius* , and *T. hiemalis* due to low sample sizes or mitochondrial genomes failing to assemble from the Southern population (which yielded *n* ≤ 5 individuals). We tested for signatures of population expansion in each species by calculating Tajima's *D* (Tajima 1989) and we and calculated nucleotide diversity for both populations using the R package pegas v 1.1 (Paradis 2010). We also created haplotype networks for ND2 using pegas.

### 2.6 Evaluating the Genomic Extent of Differentiation and Shared Patterns of Divergence

For each species, we calculated genome‐wide pairwise *F*_ST_ and created Manhattan plots to evaluate patterns of differentiation across the genome between assigned Northern and Southern populations. We generated population‐level SAF files in ANGSD using the variant sites file generated for population genetics methods. Using winsfs, we then generated a folded 2D SFS for each species. We estimated pairwise *F*_ST_ across 50 kb sliding windows with a window step of 10 kb using the stats2 function in ANGSD. We used a conservative approach commonly used to identify *F*_ST_ window outliers that were 5 standard deviations above the global *F*_ST_ mean (e.g., Walsh et al. 2019; Moreira and Smith 2023). We then visualized genome‐wide patterns of divergence by creating Manhattan plots for each species using the function “manhattan” in the R package qqman v.0.1.9 (Turner 2018).

We also tested for shared signatures of elevated genetic differentiation associated with population differences in allele frequencies by identifying shared *F*_ST_ outlier windows. Owing to the need to use the same reference genome for this analysis (Fraser and Whiting 2020), we restricted this test of molecular parallelism to groups of species that are all mapped to the same reference genome (i.e., four warbler species mapped to the *Setophaga coronata* reference genome (Toews et al. 2016), two thrush species mapped to the *Catharus ustulatus* reference genome (Louder et al. 2024), and three species from unique families but all mapped to the *Certhia americana* reference genome (Manthey et al. 2021); see Table S2). We estimated pairwise *F*_ST_ across 10 kb non‐overlapping windows using the stats2 function in ANGSD and then identified outliers that were 5 standard deviations above the global *F*_ST_ mean. We identified the positions of overlapping outlier windows between any two species that were mapped to the same reference genome. For species mapped to the *S. coronata* and *C. americana* reference genomes (Baiz et al. 2021; Manthey et al. 2021), annotations were constructed using MAKER (Cantarel et al. 2008); we therefore obtained the NCBI ID associated with the proteins with the highest confidence in each annotation. We then used the NCBI ID to identify the gene or protein name, description, and function using the NCBI database (https://www.ncbi.nlm.nih.gov/). For species mapped to the *C. ustulatus* reference genome (Louder et al. 2024), the annotation was constructed using the NCBI Eukaryotic Genome Annotation Pipeline, such that the annotation had gene ID information provided. We used the NCBI database to collect any additional information about gene function.

## 3 Results

### 3.1 Genome Sequencing

Following filtering, samples across all 12 species had a mean of 5.0× coverage across the genome (range = approximately 0.91× to 23.5× coverage). The mean mapping rate across all samples used in analyses was 89.3% (range = 53.0%–98.4%) (Table S1).

### 3.2 Population Genetics

We observed concordant patterns of spatial genetic differentiation between the boreal (“Northern”) and Appalachian (“Southern”) populations on genome‐wide PCA plots in 11 out of 12 of the species (all species except the woodpecker, *S. varius* , which did not exhibit genetic differentiation between boreal and Appalachian populations; Figure 2). Our dataset, however, contained very few southern Appalachian samples (*n* = 3) and no central Appalachian samples from *S. varius* , due to the paucity of available genetic samples, which could explain the lack of spatial genetic pattern detected. Admixture analyses further supported *K* = 2 as the best fit for the number of ancestry groups and indicated distinct differences between the Southern and Northern populations. The geographic break between these Southern and Northern clusters was most typically observed between the northern Appalachian sampling region (Pennsylvania/Ohio) and the central Appalachian sampling region (West Virginia/northern Virginia; Figures 1 and 2). We note, however, that nuclear genetic differentiation between Southern and Northern populations is very low across all species (*F*_ST_ ≤ 0.01; Table 1), indicating shallow divergence.

### 3.3 Genetic Diversity

Pairwise *θ*_*π*_ did not significantly differ between Northern and Southern populations in 6 out of 10 species included in the analyses (Table 1). In three species ( *Setophaga fusca* , *Regulus satrapa* , and *Vireo solitarius* ), the Southern population exhibited significantly higher genetic diversity (pairwise *θ*_*π*_) than the Northern population, whereas in one species ( *Setophaga virens* ), the Northern population exhibited significantly higher genetic diversity than the Southern population.

### 3.4 Mitochondrial Analyses

In the mitochondrial gene (ND2), we detected little to no population differentiation (*F*_ST_ ≤ 0.09), and low levels of Nei's genetic distance (Nei's *D* ≤ 0.017) between Southern and Northern populations. There was no observable population clustering in the haplotype networks (Figure S2). We found little nucleotide diversity across species (*π* ≤ 0.005, except for *T. hiemalis* ), and differences in diversity between populations were not detected in mitochondrial analyses. We also observed negative Tajima's *D* for all 12 species, which is consistent with the expectations for populations with recent demographic expansion (Table 1).

### 3.5 The Genomic Extent of Differentiation and Shared Molecular Patterns of Divergence

**FIGURE 3:**
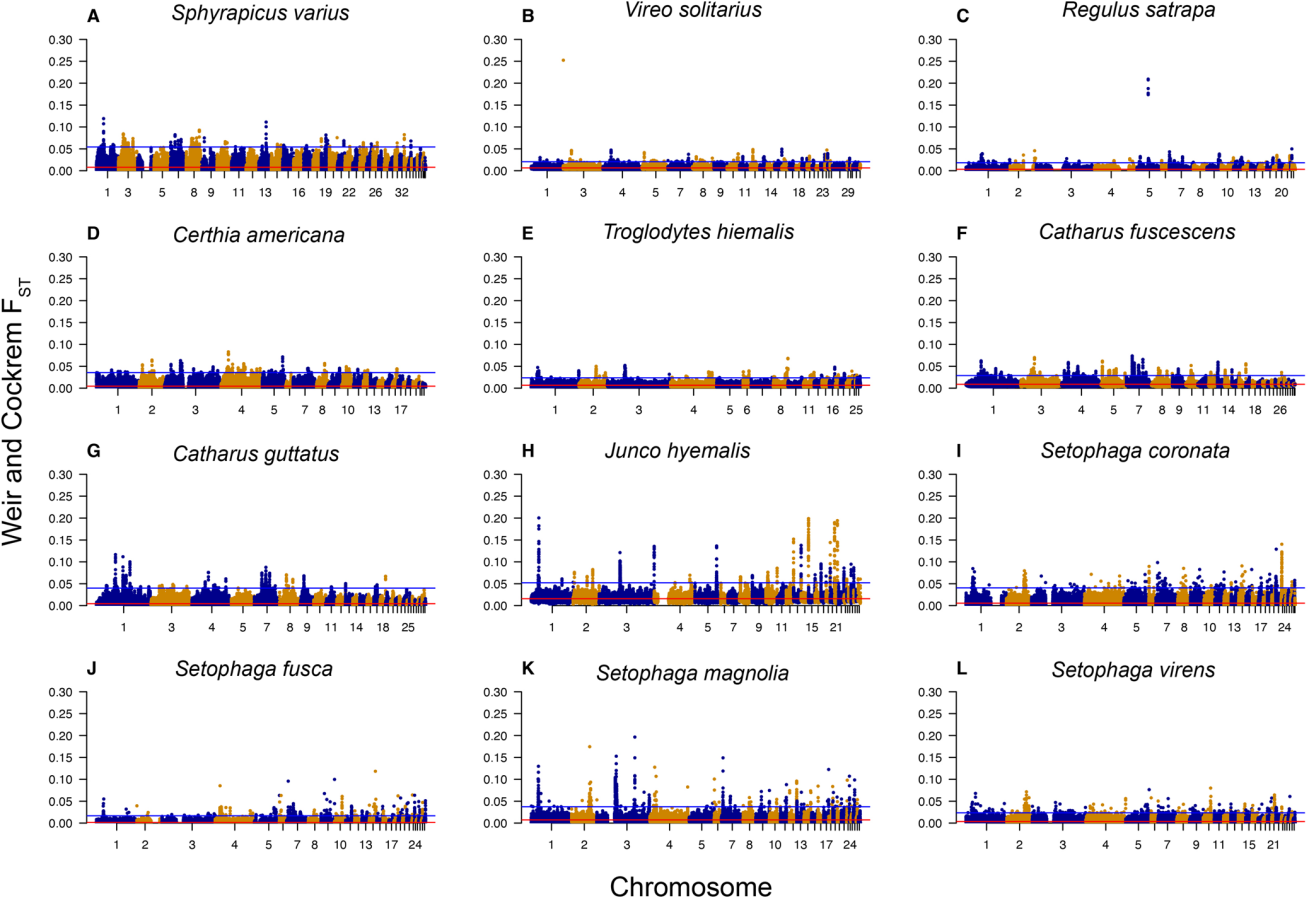
Manhattan plots of pairwise *F*_ST_ between the Southern and Northern population across 50 kb sliding windows with a window step of 10 kb calculated in ANGSD. Alternating navy and gold colors differentiate chromosomes. The orange line demarcates the global mean *F*_ST_, and the blue line demarcates five standard deviations above the global mean.

By characterizing regions of elevated differentiation across the genome, we detected outlier windows ranging from 203 windows in *S. varius* to 519 in *Setophaga magnolia* . Outlier windows accounted for an average of 0.4% of the sampled windows of each species (Figure 3). Outlier windows also exhibited low levels of differentiation (*F*_ST_ average = 0.04; range = 0.01–0.25) and were not concentrated in any specific genomic regions.

We did not identify any shared windows of elevated differences across all species in either set that included greater than 2 species (i.e., four parulid species, and three species mapped to *C. americana* ). Considering only shared outlier windows containing annotated proteins, we identified a total of 20 candidate genes for selection between the Southern and Northern populations shared by two or three parulid species. Only one candidate gene was shared by three species ( *S. fusca* , *S. magnolia* , *and S. virens*), ATP6V0B (ATPase H+ transporting V0 subunit b), which is a gene involved in proton transmembrane transport (Table S5). The candidate genes shared between two species are also primarily involved in cellular functioning, including development, maintenance, and inflammatory response (Table S5). We identified one candidate gene between Southern and Northern populations in the *R. satrapa* and *T. hiemalis* that plays a role in cell adhesion and migration (the ITGB2 gene, or integrin subunit beta 2). In the pair of *Catharus* species, we also identified only one candidate gene shared by both species, IGFBP7 (insulin like growth factor binding protein 7), which plays a role in regulating cell growth.

## 4 Discussion

We detected spatially concordant genetic divergence between the Southern (Appalachian) and Northern (boreal) populations of 11 out of the 12 sympatrically breeding birds we analyzed (Figure 2). Identification of genetically distinct populations using whole genome sequencing can be used to assign intraspecific conservation units, or designated groups used to inform management decisions to conserve species‐level genetic diversity (DeSaix et al. 2023; Paetkau 1999). By considering shared patterns of genetic population differentiation across several species, conservation efforts can prevent genetic diversity loss while taking a more inclusive community rather than species‐level approach. In our study of a co‐distributed species assemblage, our results consistently point to small populations persisting in the southern Appalachian Mountains as harboring genetic diversity that is distinct from much larger, widespread boreal populations of the same species. The degree of genetic structure, however, was subtle, as genetic differentiation between the boreal and Appalachian populations was low across all species at the level of the nuclear genome (*F*_ST_ ≤ 0.01) and a mitochondrial gene, ND2 (*F*_ST_ ≤ 0.09).

Our results detecting genetic structure between northern and southern populations are similar to those in previous studies of boreal‐Appalachian migratory bird species, including *Cardellina canadensis* (Miller et al. 2024) and *C. fuscescens* (Kimmitt et al. 2024a; a species also included in our current study). By contrast, a previous study of another warbler species, *Setophaga caerulescens* , indicated low genetic differentiation between boreal and Appalachian populations based on microsatellite and mitochondrial DNA (Davis et al. 2006). Mitochondrial DNA in our study was likewise unable to recover genetic divergence across this region, showing low divergence and no population‐level structure in the haplotype networks in all 12 species. Therefore, the subtle spatial structure we observe between boreal and Appalachian populations may require whole genome data to detect.

The concordant phylogeographic breaks we detected are unlikely explained by a specific geographic barrier. Although the population abundance of our study species may be lower in the high elevation habitat of southern Pennsylvania's Valley and Ridge geologic province (Fink et al. 2024), near where the phylogeographic break occurs in most species, these species are nevertheless continuously distributed throughout the Appalachians. And yet, concordant patterns of discrete structure between southern and central Appalachian populations suggest restricted gene flow between these regions. We argue that this reduced gene flow may be driven by philopatry and breeding site fidelity, whereby migratory birds, despite flying long distances to their nonbreeding locations, tend to return to the same geographic region and habitat type as where they were fledged or bred in a previous year (Conklin et al. 2024; Pegan et al. 2025b; Winger et al. 2019). That is, despite their mobility, migratory species may have lower than expected dispersal between these ecogeographic regions, leading to genetically distinct populations in the Appalachians, a region of great conservation concern.

We also tested whether genetic diversity patterns reflected either (1) historic range expansions out of southern refugia that resulted in higher diversity in southern populations or (2) contemporary differences in geographic area and census population size that resulted in lower diversity in southern populations. We did not detect consistent population differences in genetic diversity across the species, as 6 out of 10 species tested did not differ in genetic diversity measures between the Southern and Northern populations. In three species ( *S. fusca* , *R. satrapa* , and *V. solitarius* ), genetic diversity in the Southern population was higher than diversity in the Northern population, suggesting that southern Appalachian populations could have acted as source populations for postglacial northern expansion in these species. By contrast, in one species ( *S. virens* ), the Northern population exhibited higher genetic diversity than the Southern population, which is more consistent with the prediction that populations isolated in small geographic regions should have lower genetic diversity (Berthier et al. 2002). However, the fact that these opposing patterns of genetic diversity were not broadly observed across our study species casts doubt on their explanatory power.

Our comparative system using whole genome techniques also provided an opportunity to evaluate the genomic context of differentiation underlying spatial structure. For all species, peaks of differentiation across the genome were relatively low (*F*_ST_ ≤ 0.25) with outlier windows spread across the genome, suggesting that concordant patterns of spatial structure emerge from species‐specific processes that likely reflect neutral genetic differentiation (Hahn 2019; Lewontin and Krakauer 1973). If selection via local adaptation is driving differentiation patterns in similar ways among co‐distributed species, then concordant phylogeographic breaks may be associated with shared patterns of molecular evolution, including the genes underlying differentiation between populations (McGaughran et al. 2022). However, we found no shared windows of elevated differentiation across the four *Setophaga* species and only detected a total of 20 candidate genes between species pairs and triplets (Table S4). Similarly, we only identified one unique potential candidate gene shared between the two *Catharus* species, as well as one unique potential candidate gene in only two out of the three species mapped to the *C. americana* reference genome (Table S4).

The lack of evidence for shared patterns of genetic differentiation in our study, along with the results of several recent comparative studies, suggests that molecular parallelism can be difficult to detect or often does not explain concordant phylogeographic patterns of population differentiation. A previous study of shared genomic differentiation in parulid warblers in the western portions of their ranges in the Canadian Rocky Mountains (which included two of our species, *S. virens* and *S. coronata* ) also did not find shared *F*_ST_ peaks between pairs of hybridizing subspecies or species (Irwin et al. 2018). The authors concluded that the pairs were experiencing differing selective forces or differing genomic responses to selection. Additionally, two recent comparative studies on passerines did not find strong support for molecular parallelism, as no candidate genes were shared between all four focal species in each study, and only 2–33 candidate genes were shared between two or three species (Recuerda et al. 2024; Walsh et al. 2019). By contrast, two woodpecker species, *Dryobates pubescens* and *Dryobates villosus* , which exhibit convergent geographic patterns of plumage coloration, shared about 12% of the outlier windows corresponding to 139 shared candidate genes for selection (Moreira and Smith 2023), providing stronger evidence for molecular parallelism compared to our study and the other studies in passerines.

The comparative nature of our study suggests caution when interpreting outlier loci as candidates for selection, given the absence of molecular parallelism detected in closely related species with similar environmental pressures. Many single‐species empirical studies report a high prevalence of outliers and conclude evidence for candidate genes under selection that might explain population differentiation (Ahrens et al. 2018; McGaughran et al. 2022). However, outlier detection methods are known to be prone to false positives (Ahrens et al. 2018), and heterogeneity in relative divergence metrics like *F*_ST_ across the genome can also be generated by intrinsic genomic architecture (Cruickshank and Hahn 2014; Thom et al. 2024). Our findings further emphasize the complexity of the mechanistic underpinnings of spatial population structure by suggesting that idiosyncratic molecular pathways among species can still produce concordant patterns of spatial population differentiation.

Collectively, our results reveal strong concordance of subtle but distinct genetic structure between the boreal and southern Appalachian populations across most of the co‐distributed migratory songbirds tested. The presence of genetically distinct populations across several species associated with adjacent but unique ecoregions can inform conservation and management efforts. The field of conservation has long debated whether conservation policy should prioritize preserving ecosystem, species, or genetic diversity (Bowen 1999). With the advances and accessibility of genomic sequencing and resources, a recent emphasis has been placed on preserving genetic diversity, as it is critical for the adaptive potential of the species (Kahilainen et al. 2014) and plays a role in community and ecosystem resilience (Stange et al. 2021). In addition to conservation genetic approaches focusing on vulnerable species for species‐level conservation (e.g., Dufresnes et al. 2023; Miller et al. 2024; Senior et al. 2022), some work has also focused on community genetics among interacting species (Schweitzer et al. 2008; Stange et al. 2021) or keystone species (Chacón‐Vargas et al. 2020; de Góes Maciel et al. 2024; Thompson et al. 2019) to evaluate impact on ecosystem resilience. Our study highlights how comparative phylogeography, a classic discipline for studying the geographic context of genetic variation (Edwards et al. 2022), could be used as a broader link between preserving genetic diversity and ecosystem management, by using evidence of concordant patterns of intraspecific conservation units among co‐occurring species to inform habitat conservation policy.

## Ethics Statement

Samples sequenced newly for this study were provided from existing natural history tissue collections. For providing permits that authorized the collection of samples contributed by the authors, we thank the United States Fish and Wildlife Service, United States Forest Service, North Carolina Wildlife Resources Commission, Ohio Division of Wildlife, Pennsylvania Game Commission, Vermont Fish and Wildlife Department, Vermont Agency of Natural Resources, and West Virginia Department of Natural Resources. Sampling protocols were approved by the University of Michigan Animal Care and Use Committee (# PRO00010608).

## Conflicts of Interest

The authors declare no conflicts of interest.

## Supporting information


**Table S2:** Reference genomes used to align samples from each species, as well as the number of SNPs analyzed in PCA, and the length of random genome subset used to estimate pairwise *θ*_*π*_.
**Table S3:** Parameters used in ANGSD to estimate metrics. Parameter definitions were summarized from the ANGSD website.
**Table S4:**. A list of chromosomes regions filtered out of the dataset due to evidence of putative inversion polymorphisms. Microchromosomes (indicated by region type) were entirely discarded, such that it is unnecessary to specify filtering range.
**Table S5:** Summary of candidate genes identified by outlier windows (50 kb sliding windows with a window step of 10 kb) from genome‐wide pairwise *F*_ST_ analysis shared between two or three species. Outlier windows were identified as 5 standard deviations above the global *F*_ST_ mean. Using the *Setophaga coronata* annotation, we identified protein matches found within candidate windows. We then identified gene name, description, and function using the NCBI IDs on the NCBI database (https://www.ncbi.nlm.nih.gov/).
**Figure S1:** (A) Map of specimen sampling locations for each species. Scientific name is at the top of each panel. Each point represents an individual, but in some cases, multiple individuals were sampled from the same location, such that points are overlapping. The species' range (orange) show the mean abundance of the species during the breeding season in 2023 from the eBird Status and Trends (Fink et al. 2024). Samples from the boreal region are displayed by purple circles, with shading corresponding to longitude. Triangles show one of three potential sampling regions associated with the Appalachian Mountains south of the boreal forest belt, which include: (1) North‐Central Appalachians (teal), (2) Central Appalachians (green), (2) Southern Appalachians (yellow). Samples from each species do not always include every sampling region from the dataset.
**Figure S2:** Haplotype networks using the ND2 gene generated using the R package pegas. The Southern population is in red, and the Northern population is in turquoise.

**Table S1:** Summary table of samples used in analyses, which includes institution, species, catalogue number, sex, collection date, locality, and latitude/longitude data for each sample. Genomic coverage (total number of bases) and the percent of filtered reads mapped are also included for every individual. Coverage is equivalent to the total bases/1e9 based on the assumption that avian genomes are approximately 1 GB. Acronyms for institution names are as follows: AMNH = American Museum of Natural History; BURG = blood samples collected by Theresa Burg; CMNH = Cleveland Museum of Natural History; CUMV = Cornell University Museum of Vertebrates; MMNH = Bell Museum of Natural History; MVZ = Museum of Vertebrate Zoology, UC Berkeley; KETT = blood samples collected by Ellen Ketterson; NCSM = North Carolina State Museum; NYSM = New York State Museum; RAM = Royal Alberta Museum; UAM = University of Alaska Museum; UMMZ = University of Michigan Museum of Zoology; USNM = National Museum of Natural History, Smithsonian Institution; UWBM = University of Washington Burke Museum.

## Data Availability

The raw sequence data used in this study has been uploaded to the NCBI Sequence Read Archive under BioProjects PRJNA1043688 (Kimmitt et al. 2024b) and PRJNA1130443 (Pegan et al. 2025a).
