# Supplementary material for: Comparative Whole Genome Phylogeography Reveals Genetic Distinctiveness of Appalachian Populations of Boreal Songbirds
Source: Evol Appl. 2025 Oct 15;18(10):e70163. doi: 10.1111/eva.70163 (PMC12528544; doi:10.1111/eva.70163)
Supplement: Supplementary file 1 — Table S2: Reference genomes used to align samples from each species, as well as the number of SNPs analyzed in PCA, and the length of random genome subset used to estimate pairwise θ π . Table S3: Parameters used in ANGSD to estimate metrics. Parameter definitions were summarized from the ANGSD website. Table S4:. A list of chromosomes regions filtered out of the dataset due to evidence of putative inversion polymorphisms. Microchromosomes (indicated by region type) were entirely discarded, such that it is unnecessary to specify filtering range. Table S5: Summary of candidate genes identified by outlier windows (50 kb sliding windows with a window step of 10 kb) from genome‐wide pairwise F ST analysis shared between two or three species. Outlier windows were identified as 5 standard deviations above the global F ST mean. Using the Setophaga coronata annotation, we identified protein matches found within candidate windows. We then identified gene name, description, and function using the NCBI IDs on the NCBI database (https://www.ncbi.nlm.nih.gov/). Figure S1: (A) Map of specimen sampling locations for each species. Scientific name is at the top of each panel. Each point represents an individual, but in some cases, multiple individuals were sampled from the same location, such that points are overlapping. The species' range (orange) show the mean abundance of the species during the breeding season in 2023 from the eBird Status and Trends (Fink et al. 2024). Samples from the boreal region are displayed by purple circles, with shading corresponding to longitude. Triangles show one of three potential sampling regions associated with the Appalachian Mountains south of the boreal forest belt, which include: (1) North‐Central Appalachians (teal), (2) Central Appalachians (green), (2) Southern Appalachians (yellow). Samples from each species do not always include every sampling region from the dataset. Figure S2: Haplotype networks using the ND2 gene generated using the R p [file EVA-18-e70163-s001.docx]

**Table S2.** Reference genomes used to align samples from each species, as well as the number of SNPs analyzed in PCA, and the length of random genome subset used to estimate pairwise θ_π_.

| **Species** | **Reference genome GenBank assembly** | **Reference genome species** | **# SNPs** | **Random genome subset length** |
| --- | --- | --- | --- | --- |
| ***Sphyrapicus varius*** | GCA_014839835.1 | *Picoides pubescens* | 17188878 | 112579609 |
| ***Vireo solitarius*** | GCA_020740725.1 | *Corvus hawaiiensis* | 19557529 | 120222334 |
| ***Regulus satrapa*** | GCA_018697195.1 | *Certhia americana* | 17033660 | 98210431 |
| ***Certhia americana*** | GCA_018697195.1 | *Certhia americana* | 10324306 | 107189761 |
| ***Troglodytes hiemalis*** | GCA_018697195.1 | *Certhia americana* | 15798497 | 101038442 |
| ***Catharus fuscescens*** | GCA_009819885.2 | *Catharus ustulatus* | 40031256 | 117514777 |
| ***Catharus guttatus*** | GCA_009819885.2 | *Catharus ustulatus* | 23428865 | 117651399 |
| ***Junco hyemalis*** | GCA_003829775.2 | *Junco hyemalis* | 11723760 | 101624383 |
| ***Setophaga coronata*** | GCA_001746935.2 | *Setophaga coronata* | 18867525 | 102716000 |
| ***Setophaga fusca*** | GCA_001746935.2 | *Setophaga coronata* | 29615876 | 102340787 |
| ***Setophaga magnolia*** | GCA_001746935.2 | *Setophaga coronata* | 30105314 | 106491022 |
| ***Setophaga virens*** | GCA_001746935.2 | *Setophaga coronata* | 11967821 | 105719440 |

**Table S3.** Parameters used in ANGSD to estimate metrics. Parameter definitions were summarized from the ANGSD website.

| Parameter | Definition | Genotype likelihoods | Site allele frequency | Individual-level heterozygosity |
| --- | --- | --- | --- | --- |
| uniqueOnly | Remove reads that have multiple best hits | 1 | 1 | 1 |
| remove_bads | Removes read with a flag above 255 | 1 | 1 | 1 |
| only_proper_pairs | Include pairs of read with both mates mapped correctly | 1 | 1 | 1 |
| minMapQ | Minimum mapQ quality | 30 | 30 | 30 |
| minQ | Minimum base quality score | 30 | 30 | 30 |
| SNP_pval | Only use sites with a p-value less than [value] | 0.05 |  |  |
| minMaf | Only use sites with a minor allele frequency above [value] | 0.05 |  |  |
| GL | Estimate genotype likelihoods | 2 | 2 | 2 |
| doMajorMinor | Define what is the major and minor allele | 1 |  |  |
| doMaf | Estimate allele frequencies | 1 |  |  |
| doGlf | Define the output file format | 2 |  |  |
| doCounts | Estimate frequencies of the different bases | 1 |  |  |
| doSaf | Estimate site allele frequency likelihoods |  | 1 | 1 |
| doHWE | Test for HWE based on genotype likelihoods | 1 |  |  |
| dumpCounts | Outputs depth of each individual | 2 |  |  |
| doDepth | Outputs distribution of sequencing depths | 1 |  |  |
| dosnpstat | Report stats without filtering sites | 1 |  |  |

**Table S4.** A list of chromosomes regions filtered out of the dataset due to evidence of putative inversion polymorphisms. Microchromosomes (indicated by region type) were entirely discarded, such that it is unnecessary to specify filtering range.

| Species | GenBank accession region name (Chromosome number):  Filter start – Filter end | Region Type |
| --- | --- | --- |
| *Sphyrapicus varius* | CM025996.1 (Chr 3): 15000000 – 40000000 | Macro |
|  | CM026000.1 (Chr 7): 15000000 – 25000000 | Macro |
|  | CM026020.1(Chr 27) | Micro |
|  | CM026025.1 (Chr 32) | Micro |
|  | CM026031.1 (Chr 38) | Micro |
| *Vireo solitarius* | CM036369.1 (Chr 24) | Micro |
|  | CM036376.1 (Chr 31) | Micro |
|  | CM036381.1 (Chr 36) | Micro |
| *Regulus satrapa* | CM031858.1 (Chr 2): 40000000 – 70000000 | Macro |
|  | CM031859.1 (Chr 3): 65000000 – 90000000 | Macro |
|  | CM031870.1 (Chr 14) | Micro |
|  | CM031872.1 (Chr 17) | Micro |
|  | CM031875.1 (Chr 21) | Micro |
|  | CM031879.1 (Chr 1A): 35000000 – 60000000 | Macro |
| *Certhia americana* | CM031858.1 (Chr 2): 50000000 – 65000000 | Macro |
|  | CM031862.1 (Chr 6): 1 – 5000000 | Macro |
| *Troglodytes hiemalis* | CM031861.1 (Chr 5): 1 – 10000000 | Macro |
|  | CM031864.1 (Chr 8) | Micro |
|  | CM031867.1 (Chr 11) | Micro |
|  | CM031868.1 (Chr 12) | Micro |
|  | CM031871.1 (Chr 15) | Micro |
| *Catharus fuscescens* | CM020358.1 (Chr 23) | Micro |
|  | CM020365.1 (Chr 30) | Micro |
|  | CM020367.1 (Chr 32) | Micro |
|  | CM020368.1 (Chr 33) | Micro |
|  | CM020369.1 (Chr 34) | Micro |
|  | CM020370.1 (Chr 35) | Micro |
|  | CM020371.1 (Chr 36) | Micro |
|  | CM020372.1 (Chr 37) | Micro |
| *Catharus guttatus* | CM020339.1 (Chr 4): 70000000 – 80000000 | Macro |
|  | CM020365.1 (Chr 30) | Micro |
|  | CM020367.1 (Chr 32) | Micro |
|  | CM020368.1 (Chr 33) | Micro |
|  | CM020369.1 (Chr 34) | Micro |
|  | CM020370.1 (Chr 35) | Micro |
|  | CM020371.1 (Chr 36) | Micro |
|  | CM020372.1 (Chr 37) | Micro |
|  | CM020373.1 (Chr 38) | Micro |
| *Junco hyemalis* | CM042576.1 (Chr 3): 10000000 – 40000000 | Macro |
|  | CM042579.1 (Chr 5): 1 – 30000000 | Macro |
|  | CM042598.1 (Chr 25) | Micro |
| *Setophaga coronata* | CM027507.1 (Chr 1): 70000000 – 100000000 | Macro |
|  | CM027508.1 (Chr 2): 40000000 – 60000000 | Macro |
|  | CM027517.1 (Chr 11): 15000000 – 20000000 | Macro |
|  | CM027530.1 (Chr 25) | Micro |
|  | CM027532.1 (Chr 27) | Micro |
| *Setophaga fusca* | CM027508.1 (Chr 2): 40000000 – 60000000 | Macro |
|  | CM027510.1 (Chr 4): 5000000 – 15000000 | Macro |
|  | CM027530.1 (Chr 25) | Micro |
|  | CM027532.1 (Chr 27) | Micro |
|  | CM027534.1 (Chr 29) | Micro |
|  | CM027536.1 (Chr 1A): 40000000 – 65000000 | Macro |
| *Setophaga magnolia* | CM027507.1 (Chr 1): 100000000 – 110000000 | Macro |
|  | CM027508.1 (Chr 2): 30000000 – 50000000 | Macro |
|  | CM027530.1 (Chr 25) | Micro |
|  | CM027532.1 (Chr 27) | Micro |
|  | CM027534.1 (Chr 29) | Micro |
| *Setophaga virens* | CM027508.1 (Chr 2): 40000000 – 60000000 | Macro |
|  | CM027511.1 (Chr 5): 1 – 10000000 | Macro |
|  | CM027530.1 (Chr 25) | Micro |
|  | CM027532.1 (Chr 27) | Micro |
|  | CM027534.1 (Chr 29) | Micro |

Table S5. Summary of candidate genes identified by outlier windows (50 kb sliding windows with a window step of 10 kb) from genome-wide pairwise F_ST_ analysis shared between two or three species. Outlier windows were identified as 5 standard deviations above the global F_ST_ mean. Using the *Setophaga coronata* annotation, we identified protein matches found within candidate windows. We then identified gene name, description, and function using the NCBI IDs on the NCBI database (<https://www.ncbi.nlm.nih.gov/>).

| Shared reference genome | Species | Chromosome | NCBI ID | Gene name | Gene description | Gene function |
| --- | --- | --- | --- | --- | --- | --- |
| *Setophaga coronata* | *S. magnolia, S. virens* | 1 | XP_030120944.1 | ST3GAL6 | ST3 beta-galactoside alpha-2,3-sialyltransferase 6 | glycolipid biosynthetic process and protein glycosylation |
|  | *S. coronata, S. magnolia* | 1a | NP_001245307.1 | TXNRD3 | thioredoxin reductase | cellular response to oxidative stress |
|  | *S. coronata, S. magnolia* | 2 | XP_002199426.3 | only protein match | kelch-like protein 38 isoform X1 | protein binding |
|  | *S. fusca, S. virens* | 2 | XP_030121905.1 | DLGAP1 | DLG associated protein 1 | cellular signaling |
|  | *S.fusca, S.magnolia* | 3 | XP_030125095.2 | only protein match | neuroendocrine convertase 2 (isoform) | unknown |
|  | *S. fusca, S. virens* | 5 | XP_030129579.1 | DISP2 | dispatched RND transporter family member 2 | cellular development |
|  | *S. fusca, S. virens* | 7 | XP_030133177.2 | only protein match | cilia- and flagella-associated protein 65 (isoform) | transmembrane protein |
|  | *S. fusca, S. magnolia, S. virens* | 8 | NP_001232246.1 | ATP6V0B | ATPase H+ transporting V0 subunit b | proton transmembrane transport |
|  | *S. coronata, S. virens* | 9 | XP_002188201.2 | TM4SF4 | transmembrane 4 L six family member 4 | membrane signal transduction |
|  | *S.fusca, S.magnolia* | 9 | XP_030136216.1 | ECEL1 | endothelin converting enzyme like 1 | protein processing |
|  | *S.fusca, S.magnolia* | 9 | XP_030136066.1 | ECE2 | endothelin converting enzyme 2 | protein processing |
|  | *S. coronata, S. magnolia* | 10 | XP_030114449.1 | PRUNE2 | prune homolog 2 with BCH domain | pyrophosphatase activity, apoptotic process |
|  | *S. coronata, S. fusca* | 12 | XP_012427878.1 | TEX264 | testis expressed 264, ER-phagy receptor | DNA repair, cellular degradation |
|  | *S. fusca, S. virens* | 15 | XP_030141464.1 | PXN | paxillin | cell migration and signaling |
|  | *S. magnolia, S. virens* | 18 | XP_030140888.1 | RAC1 | Rac family small GTPase 1 | actin filament and cortical cytoskeleton organization |
|  | *S. coronata, S. virens* | 20 | XP_030122516.2 | only protein match | fer-1-like protein 6 (isoform) | membrane organization |
|  | *S.fusca, S.magnolia* | 22 | XP_030145496.1 | LOC100218400 | cytosolic purine 5'-nucleotidase (and isoform X3) | 5'-nucleotidase activity |
|  | *S.fusca, S.magnolia* | 22 | XP_002195600.1 | IL1B | interleukin 1 beta | cytokine activity, interleukin-1 receptor binding |
|  | *S.fusca, S.magnolia* | 22 | XP_030145935.1 | OGDH | oxoglutarate dehydrogenase | tricarboxylic acid cycle |
|  | *S.fusca, S.magnolia* | 26 | XP_030133607.1 | PPIL3 | peptidylprolyl isomerase like 3 | protein folding |
| *Catharus ustulatus* | *C. guttatus, C. fuscescens* | 5 | XM_033059818 | IGFBP7 | insulin like growth factor binding protein 7 | Regulation of cell growth |
| *Certhia americana* | *R. satrapa, T. hiemalis* | 7 | XP_015490935.1 | ITGB2 | integrin subunit beta 2 | Cell adhesion and migration |

Figure S1. (A) Map of specimen sampling locations for each species. Scientific name is at the top of each panel. Each point represents an individual, but in some cases, multiple individuals were sampled from the same location, such that points are overlapping. The species’ range (orange) show the mean abundance of the species during the breeding season in 2023 from the eBird Status and Trends (Fink et al. 2024). Samples from the boreal region are displayed by purple circles, with shading corresponding to longitude. Triangles show one of three potential sampling regions associated with the Appalachian Mountains south of the boreal forest belt, which include: (1) North-Central Appalachians (teal), (2) Central Appalachians (green), (2) Southern Appalachians (yellow). Samples from each species do not always include every sampling region from the dataset.

Figure S2. Haplotype networks using the ND2 gene generated using the R package pegas. The Southern population is in red, and the Northern population is in turquoise.

References

Fink, D., T. Auer, A. Johnston, M. Strimas-Mackey, S. Ligocki, O. Robinson, W. Hochachka, L. Jaromczyk, C. Crowley, K. Dunham, A. Stillman, C. Davis, M. Stokowski, P. Sharma, V. Pantoja, D. Burgin, P. Crowe, M. Bell, S. Ray, I. Davies, V. Ruiz-Gutierrez, C. Wood, and A. Rodewald. 2024. eBird Status and Trends, Data Version: 2023; Released: 2025. Cornell Lab of Ornithology, Ithaca, New York.
